# Stepwise Thermo-Responsive Amino Acid-Derived Triblock Vinyl Polymers: ATRP Synthesis of Polymers, Aggregation, and Gelation Properties via Flower-Like Micelle Formation

**DOI:** 10.3390/ma11030424

**Published:** 2018-03-15

**Authors:** Nobuyuki Higashi, Sho Matsubara, Shin-nosuke Nishimura, Tomoyuki Koga

**Affiliations:** Department of Molecular Chemistry and Biochemistry, Faculty of Science and Engineering, Doshisha University, Kyotanabe, Kyoto 610-0321, Japan; sho.matsubara68@gmail.com (S.M.); euq1704@mail4.doshisha.ac.jp (S.N.)

**Keywords:** amino acid–based vinyl polymer, ABA-type triblock copolymer, flower-like micelle, LCST, self-healing, injectable hydrogel

## Abstract

Novel thermo-responsive ABA-type triblock copolymers (poly(NAAMe*_n_*-*b*-NAGMe*_240_*-*b*-NAAMe*_n_*), *n* = 18–72) composed of naturally occurring amino acid–based vinyl polymer blocks such as poly(*N*-acryloyl-l-alanine methyl ester (poly(NAAMe)) as the A segment and poly(*N*-acryloyl-glycine methylester)(poly(NAGMe)) as the B segment have been synthesized by the atom transfer radical polymerization (ATRP). Their thermal behaviors were analyzed in dilute aqueous solutions by turbidimetry. The turbidity curves provided two-step LCST transitions, and a flower-like micelle formation was confirmed at the temperature region between the first and second LCST transitions by dynamic light scattering, AFM and TEM. At higher copolymer concentrations, hydrogels were obtained at temperatures above the first LCST due to network formation induced with the flower-like micelles as cross-linker. The hydrogels were found to be switched to a sol state when cooled below the first LCST. These hydrogels also exhibited self-healable and injectable capabilities, which were evaluated by rheological measurements.

## 1. Introduction

There is much interest in thermo-responsive water-soluble polymers exhibiting a lower critical solution temperature (LCST) due to their potential for biomedical applications such as drug delivery and tissue engineering [1]. While the most studied polymer is poly(*N*-isopropylacrylamide) (PNIPAM) [2,3,4,5], there is an increasing interest in the development of alternative thermo-responsive polymers due to concerns regarding the toxicity of the NIPAM monomer as well as the effect of PNIPAM extracts on biological system such as cells [6]. Poly[oligo(ethylene glycol) methacrylate] and its derivatives have attracted interest as an alternative to PNIPAM due to their nontoxicity and immunological properties [7,8]. Recently, we have also found that naturally occurring amino acid–based vinyl polymers show tunable LCST/UCST (upper critical solution temperature) behaviors in water, depending on the sort of amino acids and chemical modification of the pendant group [9]. The first report on amino acid based vinyl polymers was made by Mori et al. who focused on polyacrylamide containing l-proline as a collagen model [10]. Agarwal et al. demonstrated UCST behavior of poly(*N*-acryloylglycine amide) based on thermally reversible hydrogen bonding of the side chain amide group [11]. Shimada et al. found out that ureido-containing copolypeptides composed of poly(l-citrulline) and poly(l-ornithine) undergo UCST-type phase transitions under physiologically relevant conditions [12]. Unlike PNIPAM and poly[oligo(ethylene glycol) methacrylate], most biological macromolecules present high chiral preference for monomers such as l-amino acids. The polymer assemblies composed of chiral amino acids provide additional factors to regulate assembling properties and their micro-environment and represent a step forward to more biocompatible materials. In fact, the polymer and copolymer brushes prepared from amino acid–based vinyl monomers were found to exhibit temperature-induced cell capture and release on them [13] and to be applicable in cell-sheet engineering [14].

The LCST, at which the polymer exhibits a transition between hydrated and dehydrated states, is an important property for applications, and varying the LCST is essential. There are various ways to vary the LCST of a given polymer. For instance, changing the polarity of the incorporated comonomer units could vary the LCST [15,16]. In our amino acid–based polymer system, in particular, widely tunable LCST behaviors have been achieved in a temperature range from 18 °C to 73 °C by using copolymers prepared from different amino acid–based monomer combinations [9]. One can also modify the LCST by exploiting some polymer chain topological effects [17,18,19,20,21,22,23]. Topology-driven enhancement in thermal stability has been observed typically in flower-like micelle formation from intramolecularly cyclized amphiphilic ABA-type block copolymers in water [17,18,19,20,21,22,23]. Upon increasing the copolymer concentration, intramolecular loops within the flower micelle dissociate in favor of chains that connect two micelles, that is, bridge chains (crosslikers). This open association with an increasing number of bridges can ultimately lead to gelation [24]. Thus, the flower-like micelles are expected to play an important role both in tuning the LCST and in facile formation of soft hydrogels having self-healing and injectable functions.

In the present study, we describe the synthesis of ABA-type triblock copolymers, composed of poly(*N*-acryloyl-l-alanine-methylester)(poly(NAAMe)) as the A segment and poly(*N*-acryloyl-glysine-methylester)(poly(NAGMe)) as the B segment, by atom transfer radical polymerization (ATRP) technique [25,26,27], and their thermal behaviors in water and gelation properties. While the LCST value of each block segment has been reported previously as was mentioned above, the ABA-type triblock copolymer solutions exhibit interestingly two-step LCST transitions upon heating, whose temperatures are not necessarily the same as those of the corresponding homopolymers. In the heating process, it is found that a flower-like micelle formation is surely confirmed. The evaluation includes turbidimetry, dynamic light scattering (DLS), atomic force microscopy (AFM), transmission electron microscopy (TEM), and fluorescence spectroscopy measurements. In addition, the characterization of hydrogels is performed by rheological measurements.

## 2. Results and Discussion

### 2.1. Design and Synthesis of ABA-Type Triblock Copolymers from Amino Acid–Based Vinyl Monomers

Novel thermo-responsive ABA-type triblock copolymers, composed of poly(NAAMe) as the A segment and poly(NAGMe) as the B segment, were designed and synthesized by ATRP living radical polymerization method. We have previously described that the similar amino acid–based diblock copolymers from poly(NAAMe) and poly(*N*-acryloyl-β-alanine methacrylate) (poly(NAβAMe)), whose LCSTs are 18 °C and 45 °C, respectively, display a unique phase transition behavior [28]; in heating process they show normal two LCST transitions derived from each polymer segment through a UCST-like transition between those LCSTs due to micelle formation. At this UCST-like transition, a core-shell type micelle has been observed, in which the core and the shell consist of dehydrated poly(NAAMe) segments and hydrated poly(NAβAMe) segments, respectively. However, such a micelle formation has been observed only within a narrow temperature range. In order to improve such a thermal stability of micelle structure over a wider temperature range, the poly(NAGMe) segment, whose LCST (73 °C) is much higher than that of poly(NAβAMe) (45 °C), was employed as the B segment in this study. Further, the ABA-type triblock structure and the relatively longer B segment are expected to help to build flower-like micelles that are likely to behave as bridge chains in soft hydrogels such as thermo-responsive injectable hydrogel.

**Scheme 1:**
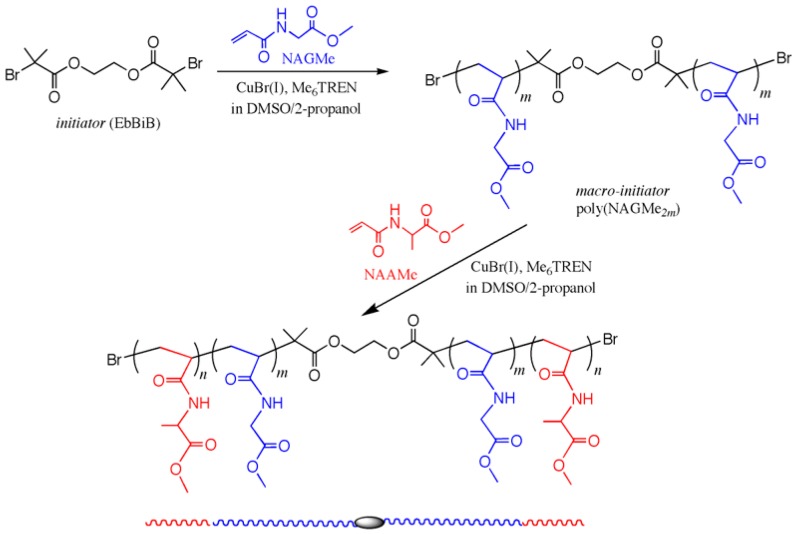
Synthetic route for the triblock copolymers poly(NAAMe*_n_*-*b*-NAGMe*_2m_*-*b*-NAAMe*_n_*) by using an atom transfer radical polymerization (ATRP) method.

Synthesis of the triblock copolymers was performed by using an atom transfer radical polymerization (ATRP) technique (Scheme 1). We first synthesized a bromine-terminated macro-initiator, composed of two poly(NAGMe) segments with the same segment length (*m*) over each other which are connected with the original initiator (EbBiB) residue. The characterization of the obtained polymer was performed by means of size exclusion chromatography (SEC) analysis and ^1^H NMR spectroscopy, and as a result the number-average molecular weight (*M*_n_) and the polydispersity index (PDI = *M*_w_/*M*_n_) were evaluated to be 34,500 (*m* = 120) and 1.19, respectively, meaning formation of a nearly monodispersed polymer. Subsequently, triblock copolymers poly(NAAMe*_n_*-*b*-NAGMe_240_-*b*-NAAMe*_n_*) were prepared by ATRP polymerization of NAAMe using poly(NAAMe_240_) as a macro-initiator. Figure S1 shows a typical SEC trace of the triblock copolymer poly(NAAMe_53_-*b*-NAGMe_240_-*b*-NAAMe_53_) obtained at a polymerization time of 30 min in comparison with the original macro-initiator (poly(NAGMe_240_)). The SEC trace of the triblock copolymer is also unimodal and shift obviously to a higher molecular weight than that of the macro-initiator, implying successful progress of the block polymerization. To examine effects of the chemical composition of the triblock copolymer on its thermal behavior, additional two triblock copolymers (poly(NAAMe*_n_*-*b*-NAGMe_240_-*b*-NAAMe*_n_*), *n* = 18 and 72), as summarized in Table S1, were synthesized by varying the polymerization time.

In view of verifying the biocompatibility of this type of triblock copolymer, the cytotoxity of poly(NAAMe_53_-*b*-NAGMe_240_-*b*-NAAMe_53_) was estimated for mouse fibroblast cells (NIH/3T3) by using the MTT assay [29]. Figure S2 displays the result for cell viability of NIH/3T3 in the presence of the triblock copolymer at concentrations of 0.1 and 1 wt %. The average viability is found to be about 90%, suggesting that the triblock copolymer does not result in serious cytotoxic effects in mouse fibroblast cells even at a concentration of 1 wt %.

### 2.2. Thermal Properties of Poly(NAAMe_n_-b-NAGMe_240_-b-NAAMe_n_) in Water at a Diluted Condition

**Figure 1:**
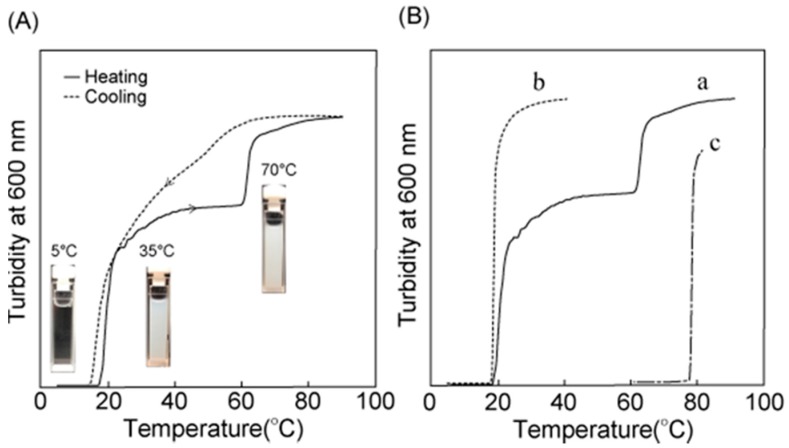
(**A**) Temperature dependence of the turbidity at 600 nm for poly(NAAMe_53_-*b*-NAGMe_240_-*b*-NAAMe_53_) aqueous solution (0.25 wt %). Heating and cooling rates are constant at 1 °C min^−1^. The insets show photographs of the solution that were acquired at the indicated temperatures in the heating process. (**B**) Comparison of the turbidity curves in the heating process between the triblock copolymer poly(NAAMe*_n_*-*b*-NAGMe_2*m*_-*b*-NAAMe*_n_*) (a) and the corresponding homopolymers of poly(NAAMe) (b) and poly(NAGMe_240_) (macro-initiator) (c).

Figure 1 shows the phase transitions observed by turbidimetry for a typical triblock copolymer poly(NAAMe_53_-*b*-NAGMe_240_-*b*-NAAMe_53_) containing the poly(NAGMe) block with about four times the degree of polymerization (DP) as the poly(NAAMe) block. Included in this figure for comparison are turbidity curves for the corresponding homopolymers of poly(NAGMe) (macro-initiator) and poly(NAAMe), which was prepared in our previous work [9]. They show single LCST transitions at 18 °C (poly(NAAMe)) and 77 °C (poly(NAGMe)). The triblock copolymer exhibits stepwise two transitions when heated. Slight hysteresis and broadening in the second transition are observed in the cooling process, while two transitions clearly appear. In the heating process, the first transition commences at almost same LCST (18 °C) as that of poly(NAAMe) homopolymer, and through a plateau the second transition takes place at around 60 °C. This temperature is, however, considerably lowered compared with the LCST value of poly(NAGMe) homopolymer (77 °C). Thus, the profile of turbidity curve for the triblock copolymer must be intrinsic and reflect an aggregation state of the triblock copolymer in water at individual temperatures.

Detailed analyses of such an aggregation state were performed by using CAC (critical aggregation concentration) and DLS evaluations, AFM, and TEM. The CAC values of the triblock copolymer were determined by using pyrene as a fluorescent probe before and after phase transitions at 5 °C, 35 °C, and 70 °C. Figure S3A shows a typical fluorescence excitation spectra recorded at 35 °C. A red-shift of the pyrene band from 333 to 336 nm was observed upon increasing the polymer concentration. This spectral shift means the transfer of pyrene from water into the hydrophobic moiety of the polymer aggregate. A plot of the ratio of the signal intensity at 336 nm to that at 333 nm (*I*_336_/*I*_333_) versus the polymer concentration (Figure S3B) resulted in a CAC value of around 7.0 × 10^−4^ wt %. The CAC value measured at 70 °C after the second transition is 2.1 × 10^−4^ wt %, which is about three times lower than that at 35 °C, probably due to easier aggregation. At 5 °C before the first transition, no concentration-dependent spectral change is observed at least below 0.1 wt %.

**Figure 2:**
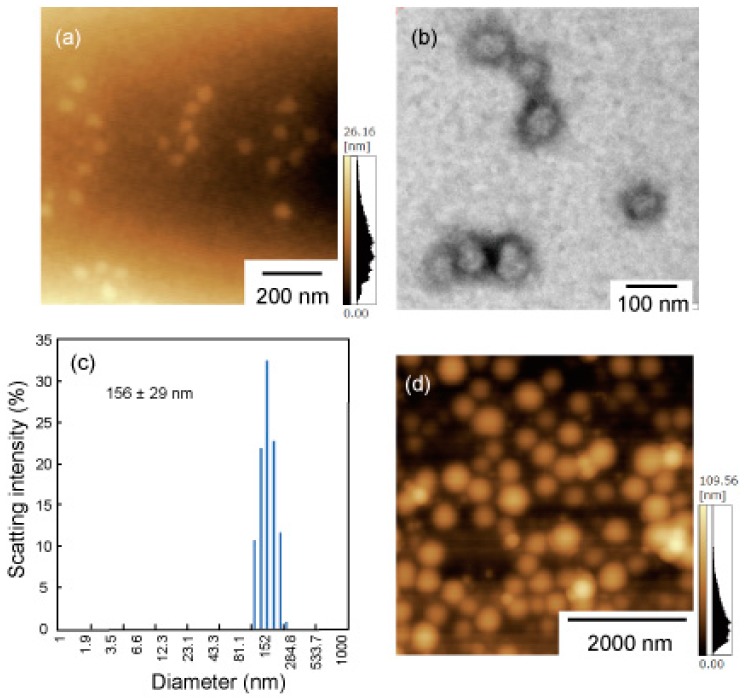
Atomic force microscopy (AFM) (**a**,**d**) and transmission electron microscopy (TEM) (**b**) images of poly(NAAMe_53_-*b*-NAGMe_240_-*b*-NAAMe_53_) aqueous solutions. Sample preparations were performed by incubation at 35 °C (**a**,**b**) and 70 °C (**d**) and subsequent staining with phosphoyungstic acid (1.0 wt %) for TEM observation. Size distribution of poly(NAAMe_53_-*b*-NAGMe_240_-*b*-NAAMe_53_) (0.25 wt %) at 35 °C was estimated by means of dynamic light scattering (DLS) measurement (**c**).

**Figure 3:**
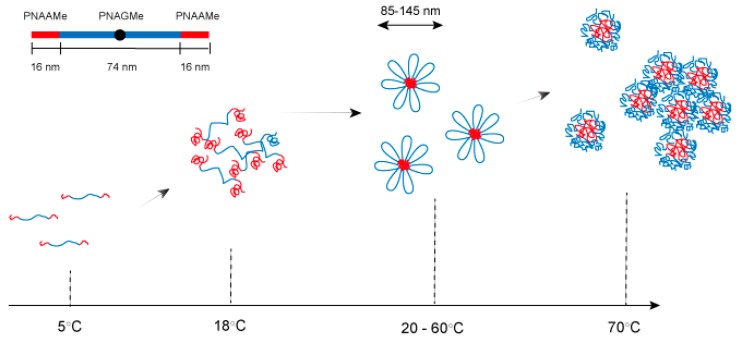
Plausible model for the thermo-responsive transitions of poly(NAAMe_53_-*b*-NAGMe_240_-*b*-NAAMe_53_) through various aggregation states containing flower-like micelle formation.

The morphological aspects for such aggregation were subsequently revealed by microscopic observations with AFM and TEM and by DLS measurement. The sample preparations were performed at the concentrations above the corresponding CAC values for these experiments. Figure 2 displays the AFM and TEM images of poly(NAAMe_53_-*b*-NAGMe_240_-*b*-NAAMe_53_) samples (0.25 wt % above CAC) prepared at different temperatures. For the sample prepared at 5 °C before the 1st transition, any marked aggregation structure did not exist in the AFM image (data not shown), suggesting that the hydrated polymer molecules disperse homogeneously. At 35 °C, which is a starting temperature of the plateau region in turbidity curve after the first LCST transition (18 °C), spherical aggregates with diameters of 85–145 nm are observed in both AFM and TEM images (Figure 2a,b). Figure 2c shows a histogram of the triblock copolymer solution at 35 °C obtained using the DLS technique. The estimated particle size 156 ± 29 nm, which is relatively close to those evaluated by AFM and TEM observations. Considering the results of microscopic and DLS observations together with the theoretical molecular length of the triblock copolymer (total length: *ca* 106 nm; A-block: *ca* 16 nm; B-block: *ca* 74 nm) estimated by assuming the most extended chain and using each DP value, it is likely that the triblock copolymer poly(NAAMe_53_-*b*-NAGMe_240_-*b*-NAAMe_53_) forms basically a flower-like micelle structure with a hydrophobic poly(NAAMe) block core and a hydrophilic, looped poly(NAGMe) block shell as illustrated in Figure 3. The hydrated triblock copolymer causes dehydration of the poly(NAAMe) block upon heating and subsequently aggregates at 18 °C. After this first LCST, the resulting amorphous aggregates are supposed to rearrange gradually to form a flower-like micelle and its loose aggregate in the plateau region of the turbidity curve. The ^1^H NMR spectra of the triblock copolymer (Figure S4A) supported such a micelle structure. The spectra were measured for 1 wt % D_2_O solution of the polymer in the presence of 4,4-dimethyl-4-silapentane-1-sulfonic acid (DSS) as an external standard at various temperatures. Figure S4B displays temperature dependences of the peak intensities for the methine proton (*a* of the molecular structure as an inset in Figure S4B) and the methylene protons (*b*), which are normalized with the peak intensity of DSS, assigned to the poly(NAAMe) block and the poly(NAGMe) block, respectively. Upon heating, the intensity of the *a* peak is reduced drastically between 15 °C and 20 °C due to dehydration of the poly(NAAMe) block and aggregate formation at the same time, corresponding to the first LCST transition. On the other hand, the intensity of the *b* peak in the poly(NAGMe) block shows almost no temperature dependence even in the first LCST transition region (15–20 °C), meaning that this block segment remained hydration state upon heating. These results strongly suggest the formation of a flower-like micelle structure with a hydrated poly(NAAMe) block shell between 20 °C and 60 °C. Further heating leads to dehydration of the poly(NAGMe) block that induces generation of huge aggregates (Figure 2d) and consequently to the second LCST transition just above 60 °C, although this transition temperature is obviously lower than that observed for the homopolymer of poly(NAGMe) (77 °C), probably due to the existence of more hydrophobic poly(NAAMe) block core.

To confirm whether the observed stepwise phase transition behavior for the triblock copolymer of poly(NAAMe_53_-*b*-NAGMe_240_-*b*-NAAMe_53_) is intrinsic or general phenomenon, two additional triblock copolymers composed of the same poly(NAGMe_240_) block and the poly(NAAMe*_n_*) block with different DPs (*n* = 18 and 72) were prepared, and their thermal properties as measured by turbidimetry were compared in Figure S5. Both polymers also show stepwise two transitions at around 20 °C (1st LCST) and 60 °C (2nd LCST), which are similar to those for poly(NAAMe_53_-*b*-NAGMe_240_-*b*-NAAMe_53_), while the extent of turbidity increment in particular at the 1st LCST transitions clearly depend on the poly(NAAMe) block length (*n*). Increasing the block length (*n*) tends to amplify the turbidity change at the first LCST transition, suggesting that elongation of the block would enhance the hydrophobic interaction working among the block segments and as a result lead to more tight aggregates. Actually, in the case of the longest block length (*n* = 72), a steep decrease in turbidity that does not appear in the case of shorter blocks (*n* = 18 and 53) is observed due to precipitation of a huge aggregate. In the case of the shortest block length (*n* = 18), the turbidity after the first LCST is found to decrease gradually until the temperature of the second LCST. We do not have any conclusive evidence so far to explain this phenomenon, but one possible explanation, which will require further exploration is as follows. The first LCST transition may occur by a tentative aggregation of the triblock copolymers due to switching the polarity of the poly(NAAMe) blocks from hydrated to dehydrated state upon heating and their subsequent hydrophobic interactions. Further increasing temperature promotes a rearrangement of the polymer molecules from amorphous aggregates to stable flower-like micelle structures. The most effective rearrangement may proceed for the polymer with the shortest poly(NAAMe) block length (*n* = 18), which is preferred for formation of the loosest aggregate at the first LCST transition. Such a smooth rearrangement in the polymer assemblies may bring about a lowering of the turbidity.

### 2.3. Self-Assembly of Triblock Copolymer in Concentrated Aqueous Solutions—Gelation and Injectable Properties

**Figure 4:**
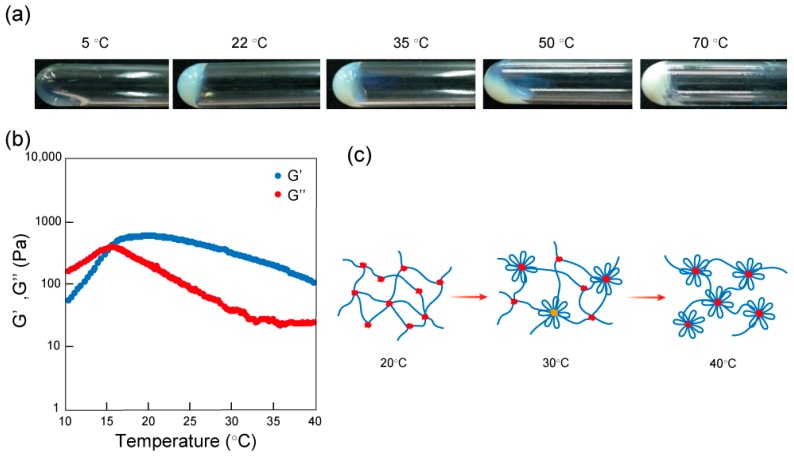
Temperature sensitivities of the hydrogels. (**a**) Photographs of poly-(NAAMe_53_-*b*-NAGMe_240_-*b*-NAAMe_53_) aqueous solutions at various temperatures. [polymer] = 30 wt %. (**b**) Temperature dependence of the storage (G’) and loss (G”) moduli for poly(NAAMe_53_-*b*-NAGMe_240_-*b*-NAAMe_53_) in the heating process from 10 °C to 40 °C at 6 rad·s^−1^, strain 1%. (**c**) Schematic illustration for a flower-like micelle network model of poly(NAAMe_53_-*b*-NAGMe_240_-*b*-NAAMe_53_) between 20 °C and 40 °C corresponding to the plateau region of the turbidity curve in Figure 1.

The self-assembly process of the triblock copolymer was further investigated in concentrated aqueous solutions (above 10 wt %). In the case of poly(NAAMe_18_-*b*-NAGMe_240_-*b*-NAAMe_18_) with a short poly(NAAMe) block, no notable gelation occurred even at a concentration of 30 wt %, while other two copolymers with longer poly(NAAMe) block formed hydrogels at 30 wt %, above 17 °C. Figure 4a displays pictures of poly(NAAMe_53_-*b*-NAGMe_240_-*b*-NAAMe_53_), a representative triblock copolymer, solution (30 wt %) at various temperatures. Above the 1st LCST transition of this triblock copolymer (18 °C), the transparent sol is transformed to the milk-white gel (22–35 °C). Upon further heating up to 50 °C, the gel is found to partially become a turbid sol and it turns again to a stiff turbid gel over the second LCST transition (70 °C). The observed fluid sol state between 35 °C and 50 °C strongly suggests the formation of a flower-like micelle structure. The rheological measurement performed for the same polymer solution (30 wt %) also supports the above discussion. Figure 4b shows temperature dependence of the storage (G’) and the loss (G”) moduli under the conditions of a frequency of 6 rad s^−1^ and 1% strain. Below 15 °C (close to the 1st LCST), the G” value is higher than that of G’, meaning that the polymer solution is in a sol state. Upon elevating temperature, the G’ value becomes reversely higher than that of G”, suggesting being in a gel state through a sol-to-gel transition. Over around 22 °C, the G’ value is found to decrease gradually, probably due to decrease in the cross-linking density in the hydrogel network although G’ always maintains the higher values than those of G” up to 40 °C. From these results it can be concluded that after the first LCST transition (15–20 °C) the hydrophobized poly(NAAMe) segments interact over each other forming a network structure with the dense cross-linking, and further increase in temperature enhances dehydration of the polymer segments and rearrangement of the cross-linking points to form a flower-like micelle as pictured in Figure 4c.

**Figure 5:**
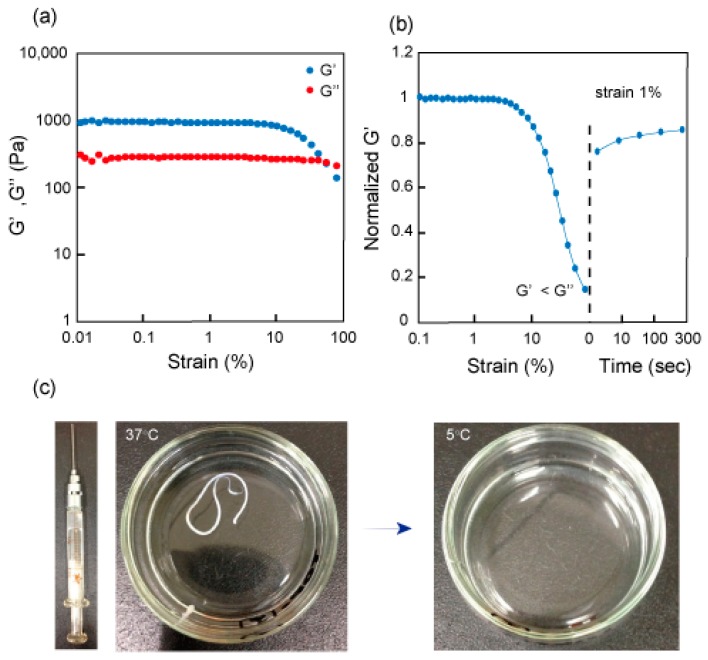
Rheology analyses of the self-healing process and injectable capability of the poly(NAAMe_53_-*b*-NAGMe_240_-*b*-NAAMe_53_) hydrogel. (**a**) Storage modulus (G’) and loss modulus (G”) on strain sweep. (**b**) Recovery of stiffness after failure incuced by shear strain. (**c**) Pictures taken when the poly(NAAMe_53_-*b*-NAGMe_240_-*b*-NAAMe_53_) hydrogel (30 wt %) was injected by using a syringe (left) into water at 37 °C (middle) and when cooled to 5 °C after one week-incubation at 37 °C (right).

Rheology measurements were also performed to trace the self-healing process for the flower-like micelle hydrogel. The G’ value decreased steeply above the critical strain region of 10% strain, indicating deformation of the gel network (Figure 5a). Thus, when the hydrogel was treated with a large amplitude strain of 100%, the G’ value decreased from ~1000 Pa to ~100 Pa, resulting in sol state with a loose network (G’ < G”). However, the G’ recover quickly to about 80% of the initial value after decreasing of the amplitude (1%) and the hydrogel returned to original state, meaning the quick recovery of the inner network (Figure 5b). Clearly, this is characteristic of a self-healing process that can be explained by the capability of the flower-like micelle gels to reform inter-micellar connections (poly(NAAMe) block and poly(NAGMe) block segments interactions) once the strain is removed.

The hydrogels that can be injected into the body, having drugs or cells that regenerate damaged tissue, would become candidates of great promise for treating many types of disease. However, these injectable gels do not always maintain their solid state once they are inside the body. Therefore, we examined whether our thermo-responsive micellar hydrogel has such an injectable capability. Figure 5c displays photographs for the injection process of the same hydrogel as used above. The hydrogel was first loaded in a syringe attached with an injection needle and cooled at 5 °C, at which in a sol (liquid-like) state. When the sol was pushed through the injection needle into a petri dish with water at 37 °C (body temperature), the original elasticity recovered in a moment and the sol returned to solid-like (gel) state whose shape had been maintained for at least one week in water at this temperature. Cooling down to 5 °C below the first LCST led the solid gel into the fluid sol due to weakening the hydrophobic interactions working among poly(NAAMe) segments and then deformation of 3D-network structure.

## 3. Materials and Methods

### 3.1. Materials

The vinyl monomers of NAAMe and NAGMe were prepared according to our previous report [9]. The initiator, ethylene bis(2-bromoisobutylrate) (EbBiB), was purchased from Sigma-Aldrich (St Louis, MO, USA) and used as received. CuBr (I) and tris [2-(dimethylamino) ethyl] amine (Me_6_TREN) for ATRP, and other solvents and materials were purchased from Wako Pure Chemical Industries (Osaka, Japan) and used as received without further purification unless otherwise noted.

### 3.2. Synthesis of Bromine-Terminated Poly(NAGMe) Macro-Initiator

A typical ATRP synthesis of the bromine-terminated poly(NAGMe_240_) macro-initiator (as the B segment) employed the following procedure. A polymerization glass tube was charged with NAGMe (1.5 g, 10.5 mmol; 3.5 M), EbBiB (12.6 mg, 35 µmol), CuBr(I) (5.0 mg, 35 µmol), Me_6_TREN (14 µL, 53 µmol), and DMSO/2-propanol (1/1 *v*/*v*). The glass tube was degassed by repeating a freeze-vaccum-N_2_ filling-thaw cycle. It was then sealed into an ampule. The polymerization was carried out at room temperature for 3 h. After removal of the Cu-complexes by passing through an alumina column with chloroform as an eluent, the condensed residue was poured into diethylether/methanol (40/1 *v*/*v*) and purified by reprecipitation with the same mixed solvent and dried. The average degree of polymerization (DP) of this macro-initiator was 240 (*m* = 120) (*M*_n_ = 34,500), determined by ^1^H NMR spectroscopy and SEC analysis (eluent: DMF; PDI (*M*_w_/*M*_n_) = 1.19).

### 3.3. Synthesis of Triblock Copolymers (Poly(NAAMe_n_-b-PNAGMe_240_-b-NAAMe_n_))

A typical protocol for the synthesis of the triblock copolymer (*n* = 53) was as follows. Poly(NAGMe_240_) macro-initiator (0.173 g, 5.0 μmol), NAAMe (1.1 g, 7.0 mmol; 3.5 M), CuBr(I) (1.4 mg, 10 μmol), and Me_6_TREN (4.1 mL, 15 μmol) were dissolved in DMSO/2-propanol (1/1 *v*/*v*). The sealed ampule was degassed by repeating a freeze-vaccum-N_2_ filling-thaw cycle, and the polymerization was carried out at room temperature for 30 min. The resulting copolymer, which was purified with the same manner as described in Section 3.2, was analyzed by ^1^H NMR spectroscopy and SEC (eluent: DMF) (*M*_n_ = 51,100, DP of NAAMe (*n*) = 53; PDI = 1.35). Other triblock copolymers with different DP (*n*) were prepared in the same manner by changing the monomer conversion.

### 3.4. Methods and Procedures

#### 3.4.1. Spectroscopies

^1^H NMR spectra for the macro-initiator and the triblock copolymers were measured at 25 °C in DMSO-*d*_6_ containing tetramethylsilan (TMS) as internal standard using a JEOL JNM-AL400 (JEOL Ltd., Tokyo, Japan) spectrometer (400 MHz). Temperature-dependence of the ^1^H NMR spectra of the triblock polymer (1 wt %) were measured in D_2_O containing DSS as external standard to investigate the thermo-induced aggregation behavior. In turbidimetry, absorption spectra were recorded at 600 nm for 0.25 wt % aqueous solutions of various polymers between 4 °C and 90 °C (heating and cooling rates: 1 °C·min^−1^) using a JASCO V-650 (JASCO Ltd., Tokyo, Japan) spectrophotometer equipped with a Peltier-type thermostat cell holder ETCS-761 (JASCO Ltd., Tokyo, Japan). To determine the critical aggregation concentration (CAC) by using a pyrene as fluorescent probe molecules, the fluorescence spectra were recorded on a JASCO FP-8300 (JASCO Ltd., Tokyo, Japan) spectrofluorometer.

#### 3.4.2. Size Exclusion Chromatography (SEC)

Molecular weights and polydispersity indices (PDI, *M*_w_/*M*_n_) of were determined by SEC using a JASCO LC-netII/AD equipped with a refractive index (RI) detector. The polymers were analyzed in DMF (containing 10 mM LiBr) (TSK-gel α-4000,Tosoh Co., Tokyo, Japan; flow rate: 0.6 mL/min, 40 °C). Poly(methymetacrylate)s (PMMA) used as the calibration standards were purchased from GL Science Inc. (Tokyo, Japan).

#### 3.4.3. MTT Assay

In order to test in vitro biocompatibility of the triblock copolymer, the MTT assay was carried out with mouse fibroblast cells (NIH/3T3). NIH/3T3 cells were cultured to 80% confluence on a dish with Dulbecco’s modified Eagle’s medium (DMEM) supplemented with 10% calf serum and 1% penicillin/streptomycin at 37 °C under 5% CO_2_. The cells were plated onto a 96-well plate at a density of ca. 1 × 10^4^ cells per well in 0.1 mL of a serum-free growth medium and incubated at 37 °C for 24 h. The growth medium was removed, and a fresh serum-free medium containing the desired amount of the block copolymer was added (final polymer concentration: 0.1, 1 wt %) and incubated for 24 h. After discarding the old media, MTT-containing medium was added to each of the wells followed by incubation for 2 h. After incubation, the media were removed, and then DMSO was added. The absorbance was measured at 570 nm in a micro-plate reader (Molecular Device Filter Max F5, Molecular Devices, San Jose, CA, USA). The cell viability (%) was calculated relative to the non-treated cells (100% viability). The experiments were performed in triplicates, in which six samples were measured per a trial.

#### 3.4.4. Microscopic Observations

The AFM images were collected at ambient temperature on a Shimadzu SPM9700 (Shimadzu Co., Kyoto, Japan) operated by tapping using a silicon tip (MPP-11100, tip radius < 12 nm, Bruker Nano Surfaces, Tucson, AZ, USA). An aliquot (~10 µL) of the aqueous solution of the block copolymer (50 µmol), which was obtained after incubation for 20 min at 35 °C, was placed on freshly cleaved mica. After adsorption for 10 s, the excess solution was removed by absorption onto filter paper, and the sample was dried at 35 °C. The scanning speed was at a line frequency of 1 Hz. The TEM images were collected on a JEOL JEM2100F (JEOL Ltd., Tokyo, Japan) at 200 kV accelerating voltage. After a small volume of the aqueous sample solution (50 µmol) was applied to a carbon-coated copper TEM grid for 20 min at a prescribed temperature, the excess solution was blotted with filter paper and stained with phosphotungstic acid (1 wt %). Finally, the sample was dried in a covered container overnight.

#### 3.4.5. Dynamic Light Scattering (DLS)

DLS measurements were performed at 35 °C by an Otsuka DLS-7000 (Otsuka Electronics Co., Ltd., Osaka, Japan) spectrophotometer equipped with a He-Ne laser (632.8 nm). The scattering light from the sample was analyzed at 90°. The non-negative constrained least-squares (NNLS) method was employed for the correction analysis to obtain the diameter probability distribution functions.

#### 3.4.6. Rheology Analyses

Rheological characterizations were performed on a Discover HR-1 (Ta Instruments, New Castle, DE, USA) equipped with a Peltie device for temperature control. During all rheological measurements, a solvent trap was used to minimize the evaporation. The measurement was carried out using a parallel plate (diameter: 20 mm). The gap was adjusted to be 400 μm to ensure that the geometry is completely filled.

## 4. Conclusions

Novel ABA-type triblock copolymers ((poly(NAAMe*_n_*-*b*-NAGMe*_240_*-*b*-NAAMe*_n_*, *n* = 18–72)) composed of amino acid–based vinyl polymer blocks were successfully prepared using the ATRP polymerization method. Turbidimetry for the diluted aqueous solutions of those copolymers showed two-step LCST transitions derived from each block segment through formation of a flower-like micelle with a hydrophobic poly(NAAMe) block core and a hydrophilic, looped poly(NAGMe) block shell. When the solution concentrations were increased, hydrogels were spontaneously formed. Rheological measurements revealed that these hydrogels exhibit sol-to-gel transition due to rearrangement of the flower-like micelles based on the LCSTs and have self-healing and injectable capabilities. Further studies of this dynamic hydrogel system are in progress to explore their application as scaffolds in cellular and tissue engineering.

## Supplementary Materials

The following are available online at http://www.mdpi.com/1996-1944/11/3/424/s1. Table S1. Characterization of the triblock copolymers by means of SEC analysis and ^1^H NMR spectroscopy; Figure S1. SEC traces of poly(NAGMe_240_) (macro-initiator) and poly(NAAMe_53_-*b*-NAGMe_240_-*b*-NAAMe_53_); Figure S2. The determination of critical aggregation concentration (CAC) of the triblock copolymer at transition temperatures by using a fluorescent dye method; Figure S3. Temperature dependent ^1^H NMR spectral change of the triblock copolymer in D_2_O; Figure S4. Comparison of the turbidity curves of triblock copolymers with different DP (*n*) of poly(NAAMe) block in water.
