# Supplementary material for: Stepwise Thermo-Responsive Amino Acid-Derived Triblock Vinyl Polymers: ATRP Synthesis of Polymers, Aggregation, and Gelation Properties via Flower-Like Micelle Formation
Source: Materials (Basel). 2018 Mar 15;11(3):424. doi: 10.3390/ma11030424 (PMC5873003; doi:10.3390/ma11030424)
Supplement: Supplementary file 1 [file materials-11-00424-s001.pdf]

Supplementary Materials

Stepwise Thermo-responsive Amino Acid-derived Triblock Vinyl Polymers: ATRP Synthesis of Polymers, Aggregation and Gelation Properties *via* Flower-like Micelle Formation

Nobuyuki Higashi[1,*] , Sho Matsubara[1], Shin-nosuke Nishimura[1] and Tomoyuki Koga[1,*]

Table S1. Characterization of poly(NAAMe$_{53}$-*b*-NAGMe$_{240}$-*b*-NAAMe$_{53}$) by means of SEC analysis and ^{1}H NMR spectroscopy.

run	Polymn. time (min)	M_n [a]	PDI [b]	$2m/n$ [a]
1	15	40100	1.25	240/18
2	30	51100	1.35	240/53
3	360	57100	1.71	240/72

[a] ^{1}H NMR, [b]SEC analysis

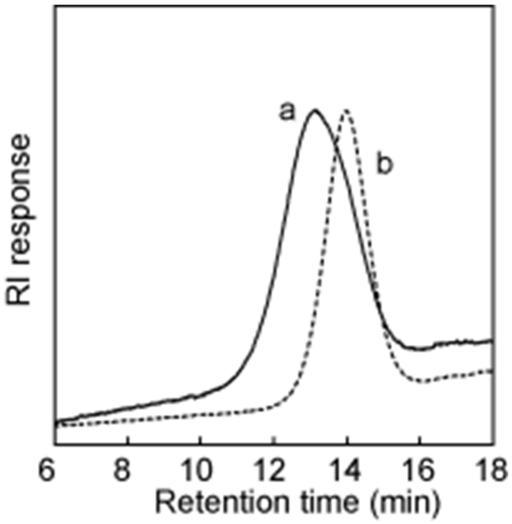

Figure S1. SEC traces of poly(NAAMe$_{53}$-*b*-NAGMe$_{240}$-*b*-NAAMe$_{53}$) (a) and poly(NAGMe$_{240}$) (macro-initiator) (b) recorded at 40 °C in DMF.

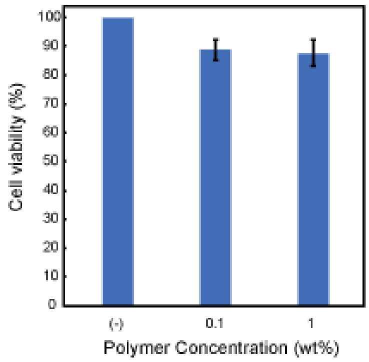

Figure S2. Viability of mouse fibroblast cells (NIH/3T3) cultured for 24 h in the presence of triblock copolymer poly(NAAMe$_{53}$-*b*-NAGMe$_{240}$-*b*-NAAMe$_{53}$) using the MTT assay. The 100% viability corresponds to the cells cultivated in the absence of the block copolymer. The experiment was performed in triplicates, in which six samples were measured per a trial.

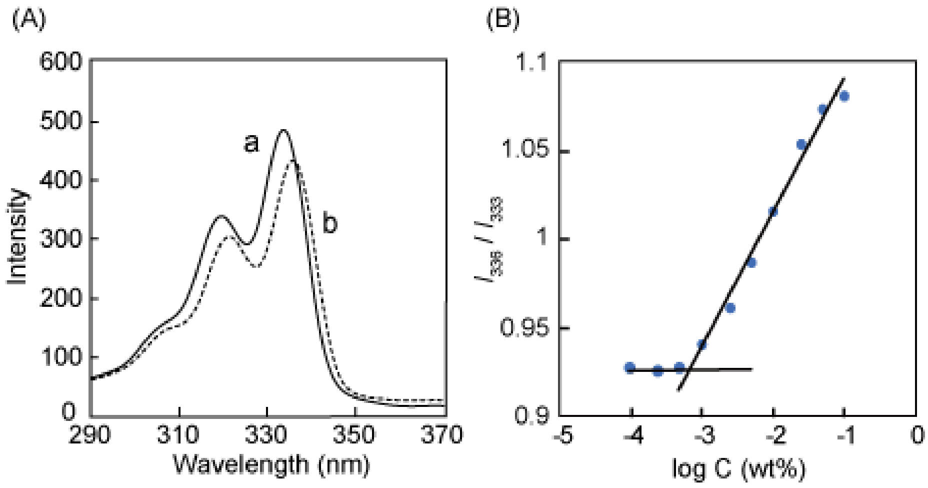

Figure S3. (A) Fluorescence excitation spectra of pyrene in poly(NAAMe$_{53}$-*b*-NAGMe$_{240}$-*b*-NAAMe$_{53}$) aqueous solutions at 1.0 x 10^{-4} wt% (a) and at 0.1 wt % (b), 35 °C. (B) Intensity ratio (I_{336}/I_{333}) obtained from the fluorescence excitation spectra of pyrene as a function of triblock copolymer concentration at 35 °C.

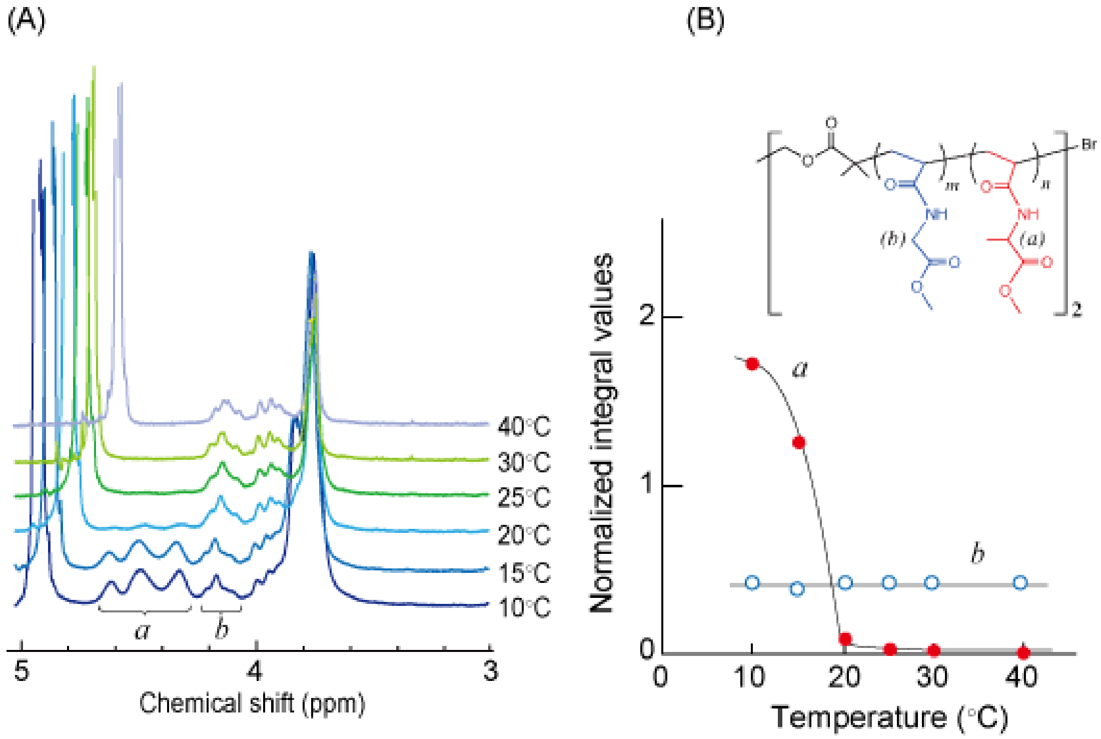

Figure S4. (A) ^{1}H NMR spectra of poly(NAAMe$_{53}$-*b*-NAGMe$_{240}$-*b*-NAAMe$_{53}$) in D_2O at different temperatures as indicated. Peaks of (*a*) and (*b*) in the spectra are assigned to the protons in the molecular structure as drawn in the inset. (B) Temperature dependences of the normalized peak intensities for such protons with PSS as an internal standard.

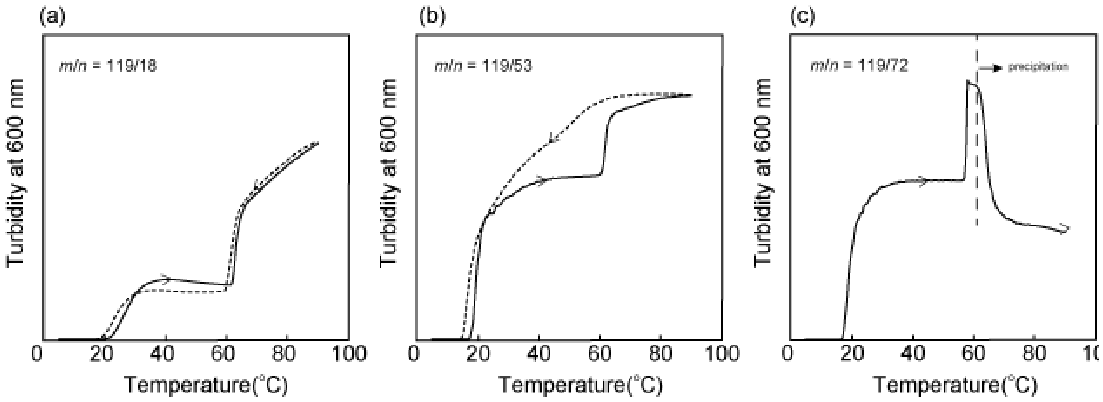

Figure S5. Comparison of the turbidity curves of triblock copolymers poly($NAAMe_{n}$ -*b*-$NAGMe_{240}$-*b*-$NAAMe_{n}$) (a: *n*=18; b: *n*=53; c: *n*=72) in water (0.25 wt %).
